# Supplementary material for: Early induction of cytokine release syndrome by rapidly generated CAR T cells in preclinical models
Source: EMBO Mol Med. 2024 Mar 21;16(4):784–804. doi: 10.1038/s44321-024-00055-9 (PMC11018744; doi:10.1038/s44321-024-00055-9)
Supplement: Supplementary file 1 — Appendix [file 44321_2024_55_MOESM1_ESM.pdf]

Rapid cytokine release syndrome caused by short-term CAR T cells in a preclinical mouse model

Appendix

Table of Content

Appendix Figures..Page

Appendix Figure S1..1

Appendix Figure S2..2

Appendix Figure S3 3

Appendix Tables

Appendix Table S1...4

Appendix Table S2 5

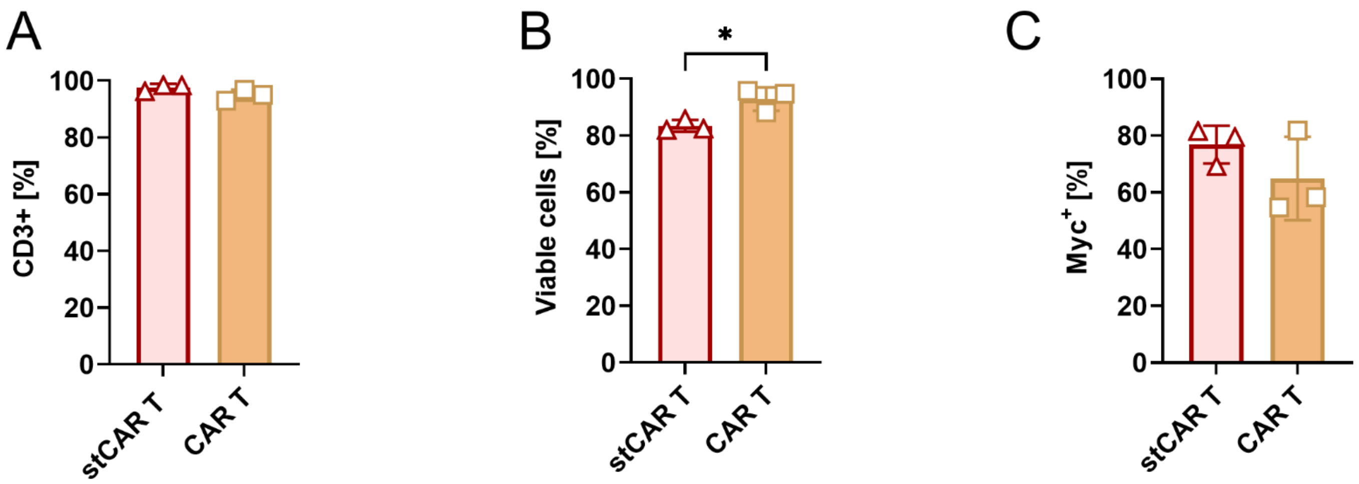

Appendix Figure S1: Properties of short-term and conventional CAR T cells.
Short-term CAR T (stCAR T) cells and CAR T cells were generated from human PBMCs. T cell frequencies **(A)** and cell viabilities **(B)** were determined by flow cytometry. Mean values and standard deviations are shown for three donors. **(C)** Expression of CAR detected after over night co-culture with NALM6 target cells as determined by myc tag detection by flow cytometry. Statistics were determined by unpaired t-test with indicated significant $p= 0.0235$.

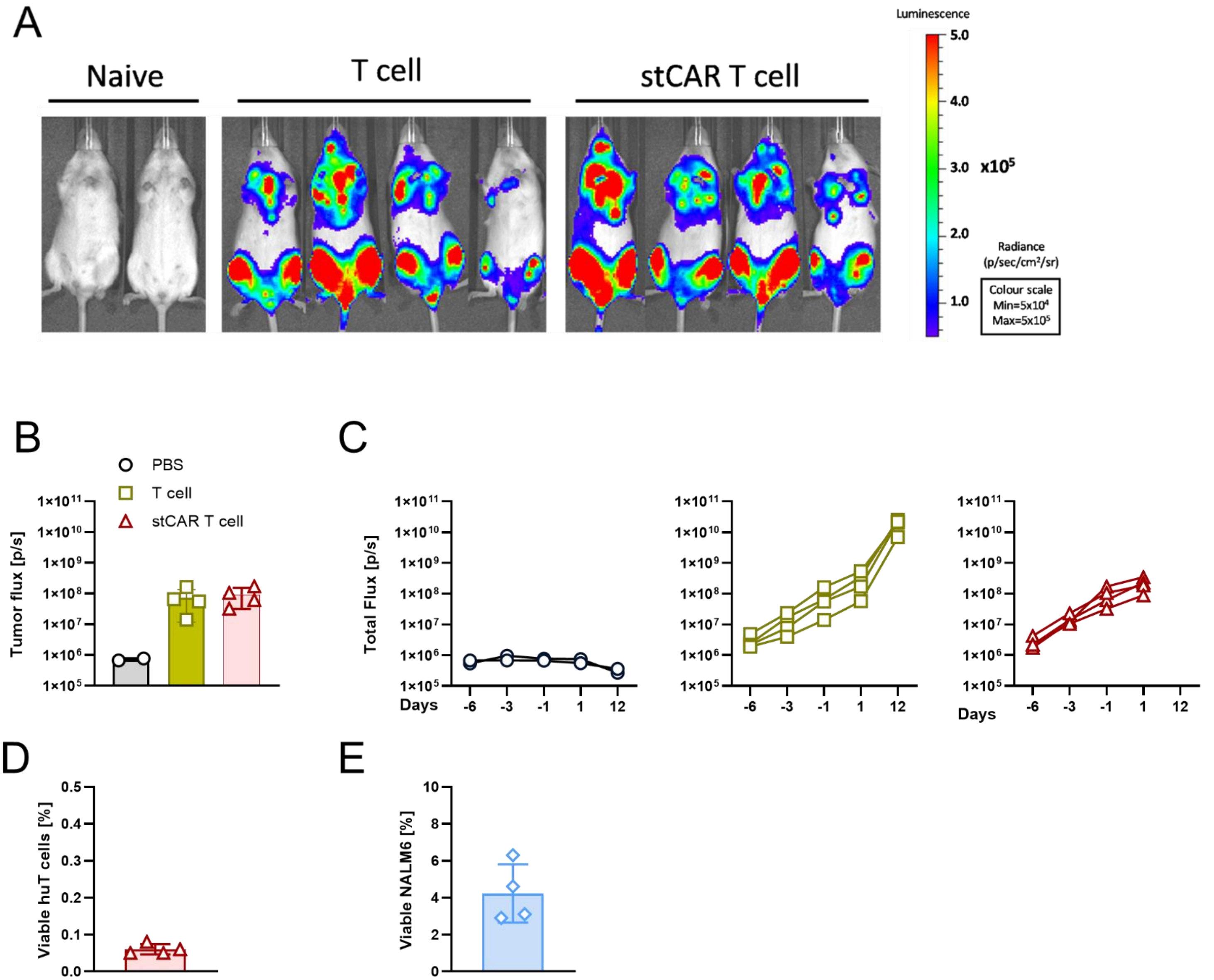

Appendix Figure S2: Tumor cell engraftment in NSG-SGM3 mice.
Tumor burden in NSG-SGM3 mice engrafted with EBFP and luciferase expressing NALM6 tumor cells was determined by *in vivo* bioluminescence imaging (IVIS). Mice without tumor cell engraftment (PBS) were used to determine background signals. **(A)** Tumor burden one day prior to stCAR T cell and T cell treatment are shown as IVIS images of the ventral side of each mouse with a color scale for tumor signal intensity. **(B)** Bar diagrams summarize quantification of the tumor flux with a single data point for each mouse including mean and standard deviation of the group. **(C)** The kinetics of the tumor flux over time is shown for each mouse represented by one line. Percentages of human T cells **(D)** and Nalm6 cells **(E)** in the bone marrow. n = 2 (PBS), 4 (T cell), 4 (stCAR T cell).

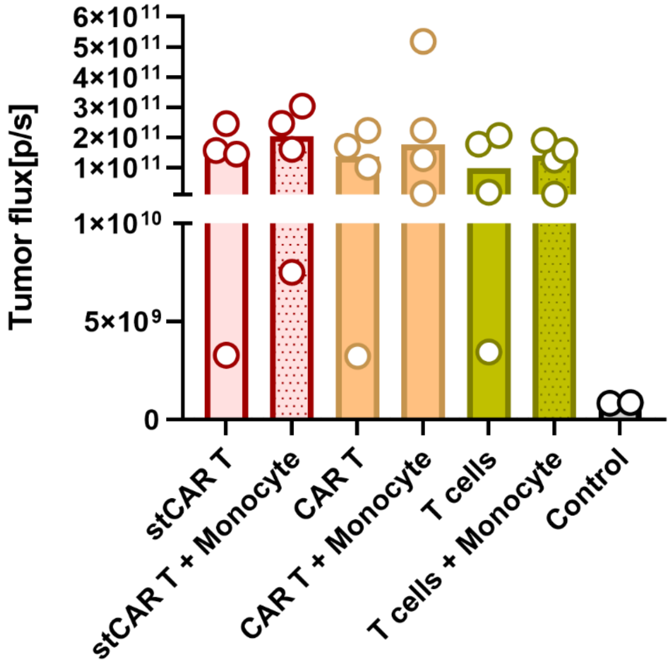

Appendix Figure S3: Supplemenatry data to Fig. 4
Tumour burden was assessed by measuring luciferase activity on day 9 after NALM6 cell injections using IVIS. Based on the measured signals, animals were assigned to the different groups to achieve equal tumour burden between groups.

Appendix Table S1: P-values of statistically significant pair-wise comparisons for the levels of neutrophils in the groups of Fig. EV3B

Comparisons	p value[1]
stCART wo target vs. stCART+mono	0.0006
stCART vs. stCART+mono	0.0056
stCART+mono vs. CART	0.0008
stCART+mono vs. CART+mono	0.0167
stCART+mono vs. T cells	<0.0001
stCART+mono vs. T cells +mono	<0.0001
stCART+mono vs. PBS	<0.0001

[1]as determined by two-way ANOVA Šídák's multiple comparisons test

Appendix Table S2: Detailled information about antibodies used in the study

	Antibody	Clone	Flurochrome	Concentration	Volume	Company	Order NO
1	**FcR-blocking**	-	-		1µL	Miltenyi Biotec	130-059-901
2	**CD45**	5B1	VioBlue	900 µg/µl	0.5µL	Miltenyi Biotec	130-092-880
		2D1	BV510	0.005 µg/µl	0.5µL	BioLegend	368526
3	**CD3**	BW264/56	PerCP	375.0 µg/µl	0.5µL	Miltenyi Biotec	130-113-131
		HIT3a	BV605	0.1 µg/µl	0.5µL	BD Bioscience	564712
5	**CD14**	REA599	APC	25µg/µl	0.5µL	Miltenyi Biotec	130-110-520
		Tuk	Percp	25µg/µl	0.5µL	Miltenyi Biotec	130-094-969
6	**CD4**	L200	PE	0.003 µg/µl	0.5µL	BD Bioscience	550630
		VIT-4	PerCp/ VioBlue	500µg/µl 200µg/µl	0.5µL	Miltenyi Biotec	130-113-779/219
7	**CD8**	RPA-T8	BV786	0.003 µg/µl	0.5µL	BD Bioscience	555369
		BW135/80	APC/PE		0.5µL	Miltenyi Biotec	130-113-154
8	**VSV-G**	8G5F11	unlabeled	0.1 µg/µl	0.1µL	Kerafest	EB0010
9	**anti-mouse IgG**	polyclonal	AF647	1.4mg/ml	0.5µL	Jackson ImmunoResearch	115-605-164
10	**CD45RA**	T6D11	VioBlue	450µg/µl	0.5µL	Miltenyi Biotec	130-113-360
11	**CD62L**	145/15	Pe-Vio 770	37.5µg/µl	0.5µL	Miltenyi Biotec	130-113-621
12	**LAG-3**	REA351	VioBlue	100µg/µl	0.5µL	Miltenyi Biotec	130-118-549
13	**PD1**	PD1.3.1.3	PE-Vio770	225µg/µl	0.5µL	Miltenyi Biotec	130-117-698
14	**TIM-3**	7D3	PE	0.1 µg/µl	1µL	BD Bioscience	563422
15	**Ly-6G**	REA126	PE-Vio770	10µg/µl	1µL	Miltenyi Biotec	130-107-977
16	**CD11b**	M1/70	Percp	0.2 µg/ml	0.5µL	eBioscience	45-0112-80
17	**CD11C**	N4/18	VioBlue	0.2 µg/ml	0.5µL	eBioscience	15-0114-82
18	**anti-Myc**	SH1-26e7.1.3	FITC	500µg/µl	0.5µL	Miltenyi Biotec	130-116-485
