# Supplementary material for: Early induction of cytokine release syndrome by rapidly generated CAR T cells in preclinical models
Source: EMBO Mol Med. 2024 Mar 21;16(4):784–804. doi: 10.1038/s44321-024-00055-9 (PMC11018744; doi:10.1038/s44321-024-00055-9)
Supplement: Supplementary file 9 — Expanded View Figures [file 44321_2024_55_MOESM9_ESM.pdf]

Expanded View Figures

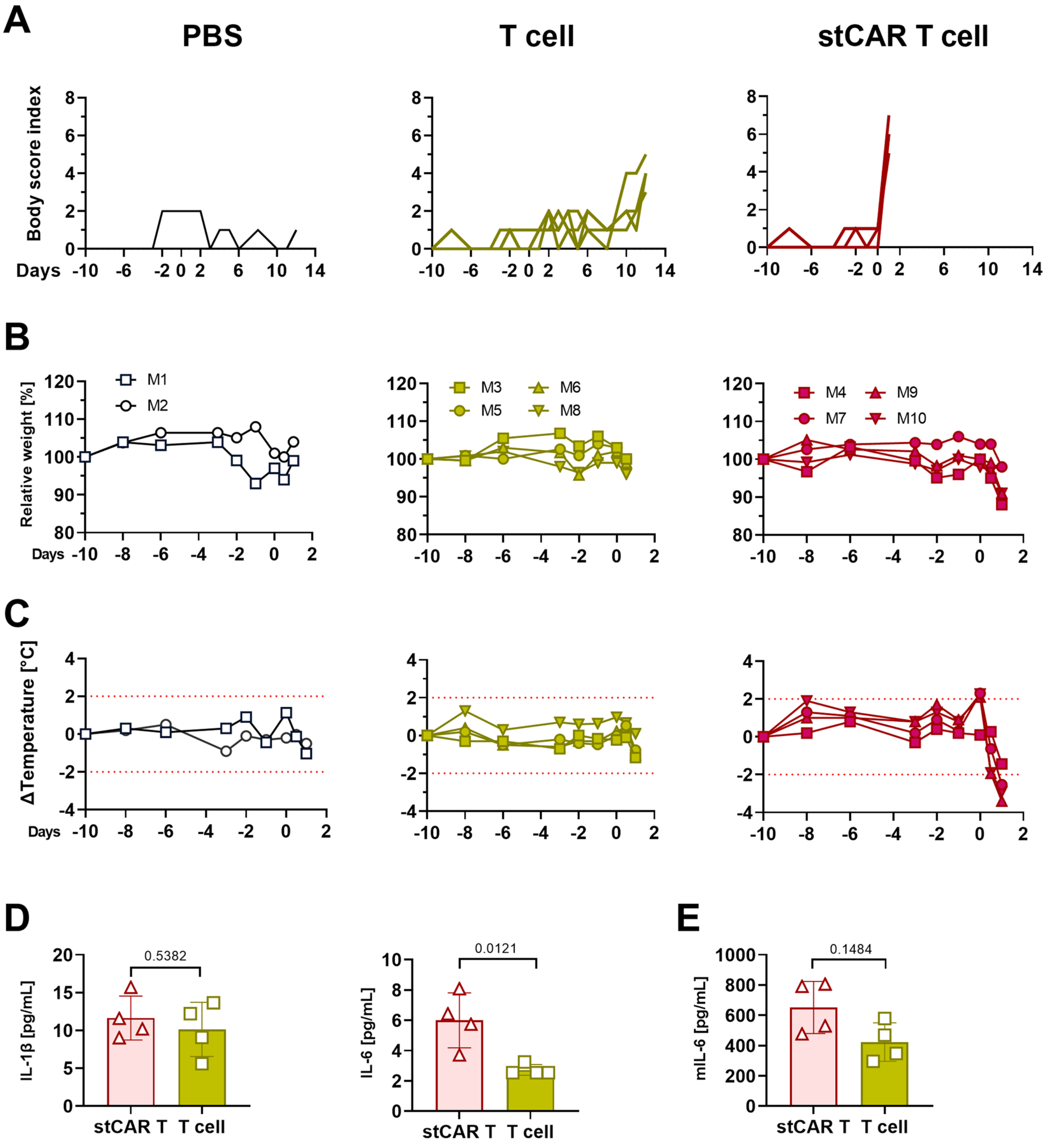

Figure EV1. Additional data to mouse study shown in Fig. 2.

(**A**) Body score indices for each mouse of the respective groups over time after cell injections. (**B**, **C**) Changes in weight and body temperature of all three groups over time. (**D**) Plasma levels of human IL-6 and IL-1β measured by multiplex kit after termination of the experiment. (**E**) Murine IL-6 level at termination day. Data information (**D**, **E**): In both groups (stCAR T cells and T cells) $n = 4$ animals. Graphs represent individual data with mean and standard deviation. Statistics were determined by unpaired t test with P values provided.

 © The Author(s)

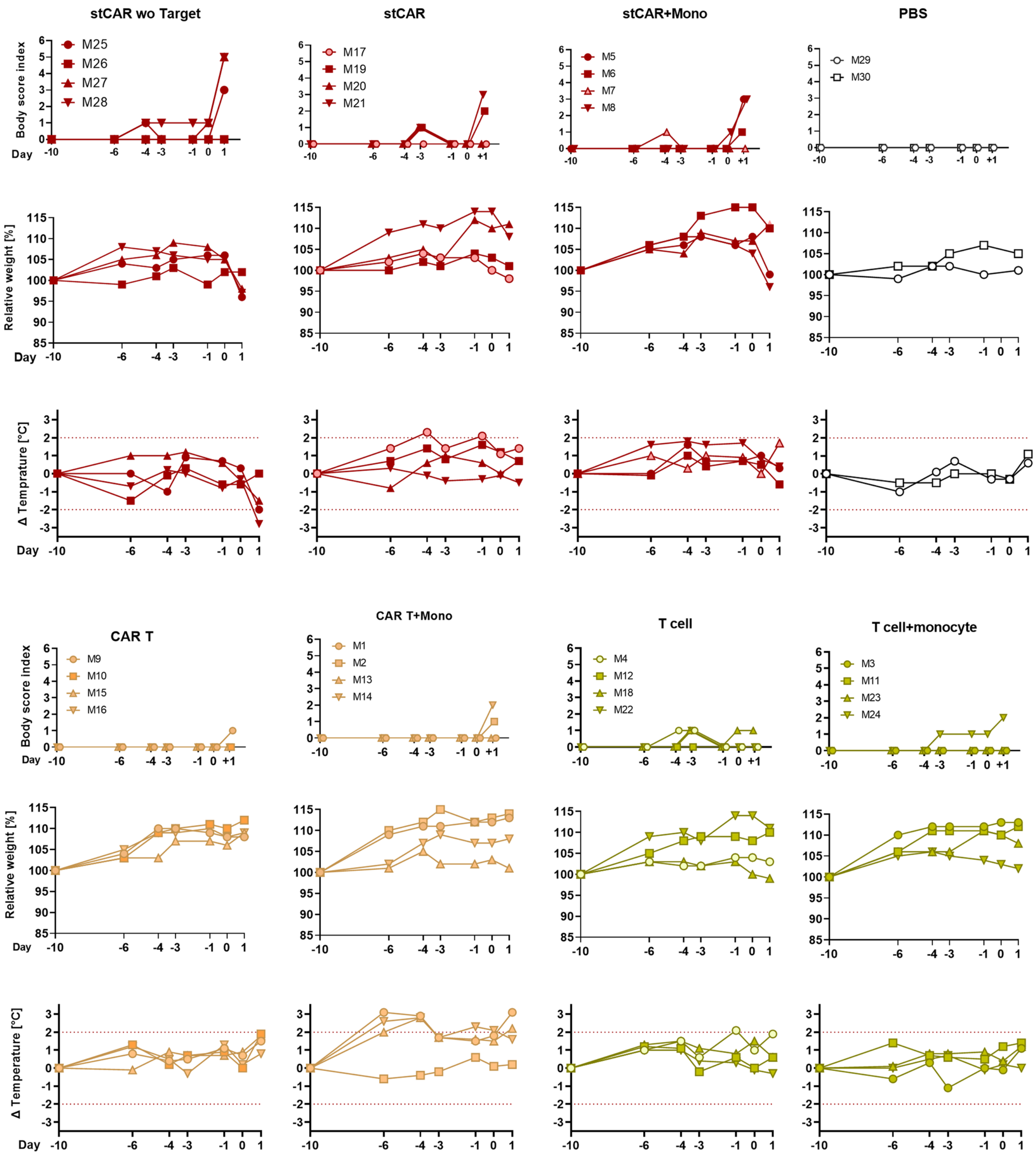

Figure EV2. Health status of individual mice from the study shown in Fig. 4.

Body score indices, body weights and temperatures of all mice included in the study are shown in Fig. 4. Individual animals from the groups having received stCAR T cells (red lines), PBS (black lines), conventional CAR T cells (yellow lines), and control T cells (green lines) are distinguished by the symbols provided in the top diagram, respectively. Individually calculated relative weights and changes in body temperature were related to the start time point of the study. Data for all eight groups are shown; $n = 4$ for all treated groups and $n = 2$ for PBS group.

© The Author(s)

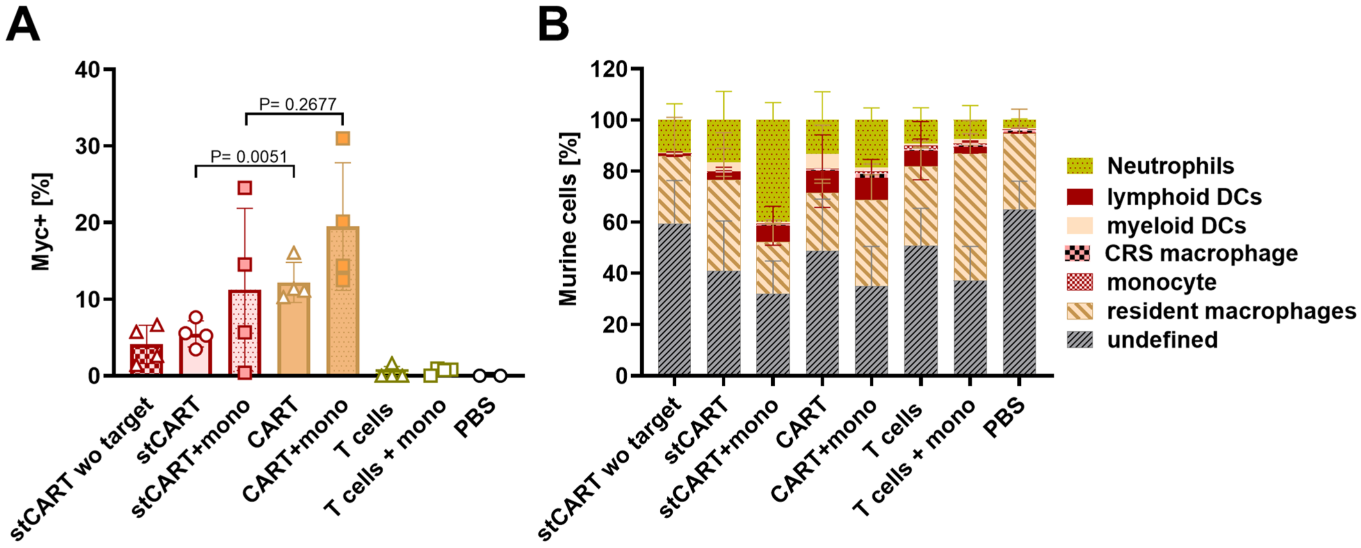

Figure EV3. Cellular analysis of mice from the study shown in Fig. 4.

(**A**) Frequencies of CAR-positive cells in bone marrow determined by flow cytometry with antibodies against the myc tag on the CAR. Data are shown for individual mice with mean values and standard deviations for the each group. Statistical significance was tested with unpaired *t* test. *P* values are provided. (**B**) Composition of murine cells in the spleen from different groups shown as stacked bars with the mean and standard deviation for each fraction of the splenocyte populations. (**A**, **B**) $n = 4$ for all treated groups and $n = 2$ for PBS group. Statistical significance of pair-wise comparisons between the levels of neutrophils in all groups are shown in Appendix Table S1.

 © The Author(s)

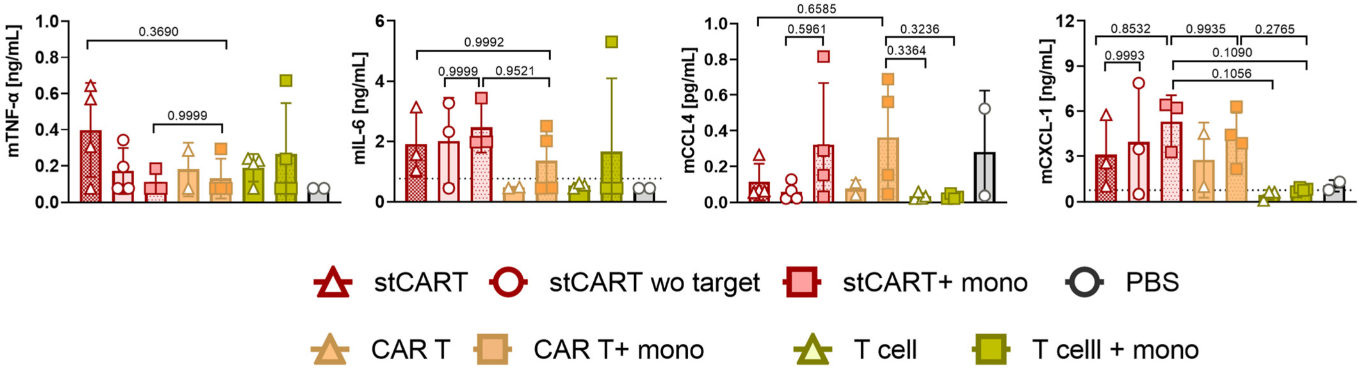

Figure EV4. Murine cytokines in mice from the study shown in Fig. 4.

Levels of the indicated cytokines in plasma obtained upon scarifying the animals. Values for each individual mouse are indicated. Bars represent mean values and standard deviations. Statistical analysis was performed by using one-way ANOVA with Tukey's multiple comparisons test to show mean differences among the groups. Mice with low tumor burden were excluded in statistical analysis (see Appendix. Fig. 3), which was not possible for CAR T-cell group ($n = 2$). P values are provided.

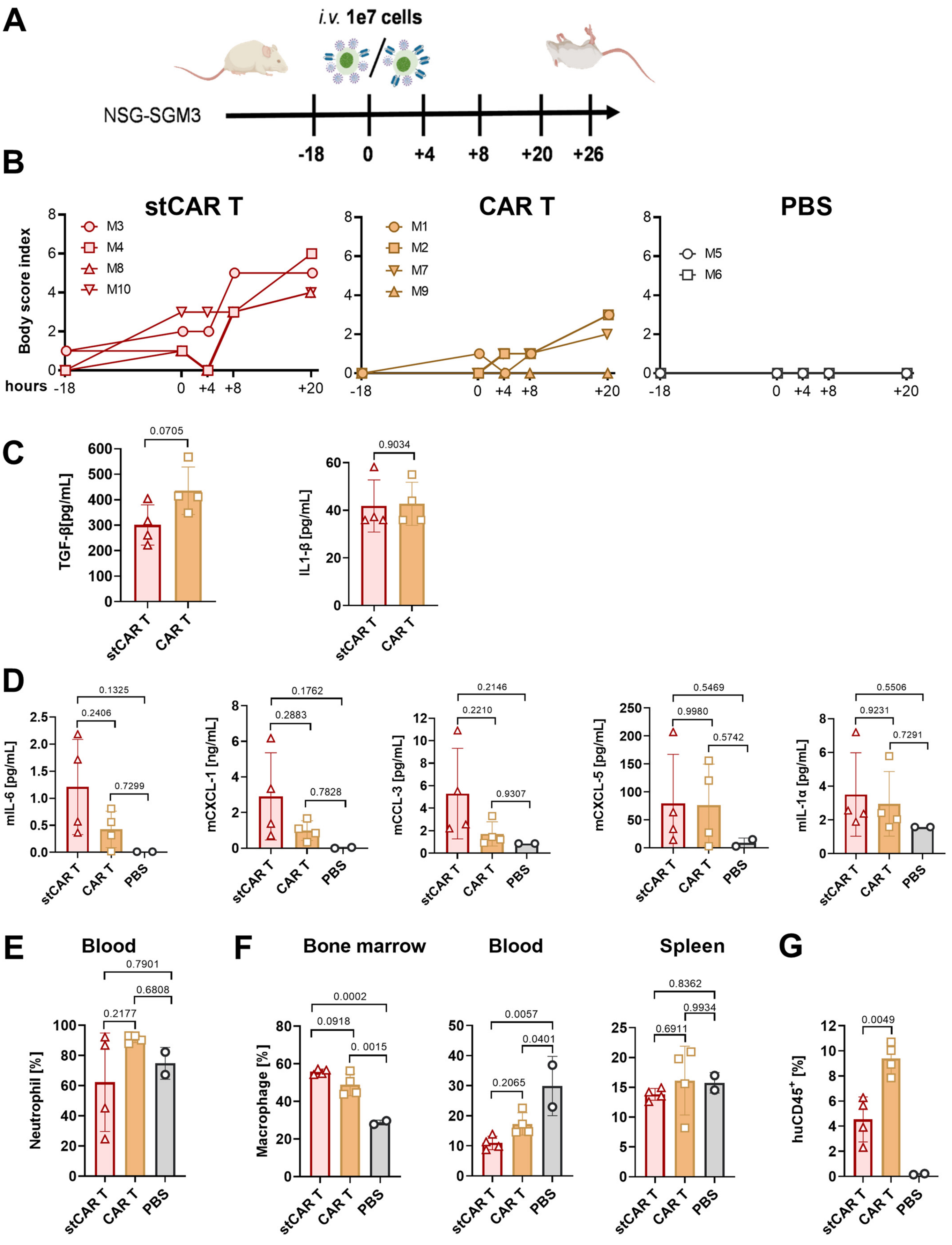
A
i.v. 1e7 cells
NSG-SGM3
-18 0 +4 +8 +20 +26
B
stCAR T
CAR T
PBS
Body score index
M3 M4 M8 M10
M1 M2 M7 M9
M5 M6
hours -18 0 +4 +8 +20
C
TGF-β[pg/mL]
0.0705
IL-1-β [pg/mL]
0.9034
stCAR T CAR T
D
mIL-6 [pg/mL]
0.1325 0.2406 0.7299
mCXCL-1 [ng/mL]
0.1762 0.2883 0.7828
mCCL-3 [pg/mL]
0.2146 0.2210 0.9307
mCXCL-5 [pg/mL]
0.5469 0.9980 0.5742
mIL-1α [pg/mL]
0.5506 0.9231 0.7291
stCAR T CAR T PBS
E
Blood
Neutrophil [%]
0.7901 0.6808 0.2177
F
Bone marrow
Macrophage [%]
0.0002 0.0918 0. 0015
Blood
0.0057 0.0401 0.2065
Spleen
0.8362 0.9934 0.6911
G
huCD45+ [%]
0.0049

© The Author(s)

Figure EV5. Supportive data to Fig. 5.

(**A**) Experimental outline. The timeline is indicating hours. (**B**) Body score indices of individual animals over time. (**C**, **D**) Cytokine concentrations of human TGF-β and IL-1β (**C**) as well as of murine IL-6, CXCL-1, CCL-3, CXCL-5, and IL-1α (**D**) in plasma collected at termination time point. (**E**) Frequencies of murine neutrophils in blood. (**F**) Frequencies of murine macrophages in bone marrow, blood, and spleen. (**G**) Human $CD45^+$ cells in bone marrow. Data information: Bars show the mean and standard deviation for each group (**C–G**). Statistics were determined by unpaired *t* test (**C**) two-way ANOVA with Tukey's multiple comparisons test (**F**). *P* values are provided.

© The Author(s)
